# Unexplained subcutaneous swelling: Keep echinococcosis in mind! Report of two primary extrahepatic subcutaneous echinococcosis cases and literature review

**DOI:** 10.1371/journal.pntd.0013511

**Published:** 2025-09-15

**Authors:** Louis Bohard, Eléonore Brumpt, Noémie Tissot, Marie Lacoste, Olivia Chapuis, Sophie Felix, Jenny Knapp, Carole Hartmann-Gouvenot, Yannick Jeffredo, Frederic Grenouillet, Damien Montange, Anne-Pauline Bellanger, Catherine Chirouze, Solange Bresson-Hadni

**Affiliations:** 1 Infectious Diseases Department, Jean Minjoz University Hospital Besançon, Besançon, France; 2 UMR CNRS Chrono environment laboratory, Marie et Louis Pasteur University, Besançon, France; 3 Visceral Radiology Department, Jean Minjoz University Hospital, Besançon, France; 4 Infectious Diseases Unit, Alpes-Léman Hospital, Contamine/Arve, France; 5 Private Pathology Laboratory, Argonay, France; 6 Pathology Laboratory, Jean Minjoz University Hospital, Besançon, France; 7 National Reference Center for Echinococcoses and FrancEchino Network, Parasitology-Mycology Laboratory, Jean Minjoz University Hospital, Besançon, France; 8 Private General Medicine Office, Belfort, France; 9 Private Pathology Laboratory, Belfort , France; 10 Medical Biology Department, Jean Minjoz University Hospital, Besançon, France; 11 Clinical Pharmacology Laboratory, Jean Minjoz University Hospital, Besançon, France; University of Liverpool, UNITED KINGDOM OF GREAT BRITAIN AND NORTHERN IRELAND

## Abstract

Alveolar and cystic echinococcoses (AE and CE) are parasitic zoonoses, mainly affecting the liver. Primary extrahepatic localizations remain rare and are difficult to diagnose. We report two cases of primary subcutaneous echinococcosis and the largest literature review on the subject. The first case is an alveolar echinococcosis located in the forehead region and the second a cystic echinococcosis in the abdominal wall. To our knowledge, a primary AE location in the face has never been described before. Pre-surgical diagnosis was not made in these two cases. However, it is essential to apply specific measures, depending on the type of echinococcosis (AE or CE), to prevent parasitic dissemination and recurrence. In view of the cases presented here, prolonged albendazole can be a second-line alternative to a surgical strategy. Echinococcosis should be kept in mind for differential diagnosis of soft tissue lesions in any part of the body.

## Author summary

Echinococcoses can appear in polymorphous clinical presentations which sometimes make diagnosis difficult, particularly in case of primary extrahepatic locations. In fact, in this situation, specific serology may not be contributory and imaging features less characteristic. However, preoperative diagnosis is essential in order to apply measures specific to these pathogens, thereby reducing the risks of treatment failure and intraoperative dissemination. Our two case reports of echinococcosis, one alveolar and one cystic along with the associated literature review focus on primary echinococcosis of the subcutaneous tissues, describing the presumed mechanism of these infections, their clinical presentation, the radiological features, the value of serology and the management modalities suggested in the literature. Most of the reported cases are cystic echinococcosis cases. Moreover, to our knowledge, we describe here the first case of primary subcutaneous alveolar echinococcosis.

## Introduction

Alveolar echinococcosis (AE) and cystic echinococcosis (CE) are zoonotic infections caused by larval stages (metacestode) of *Echinococcus multilocularis* and *Echinococcus granulosus,* respectively [1]. While CE is cosmopolitan, AE is rarer and limited to the Northern hemisphere, but the latest epidemiological data indicate an increasing incidence [2,3]. The two diseases are caused by infection with parasites of the Taeniidae family which have different transmission cycles and different clinical presentations. However, both progress slowly in the liver, the usual primary infection site [2,3]. In CE, the lesion (metacestode) is cystic and well-defined. Its peripheral layer corresponds to the adventitial layer, resulting from a fibro-inflammatory host reaction [1], whereas in AE, the lesion is an infiltrative heterogeneous lesion surrounded by an intense granulomatous and fibrous host reaction. We describe two uncommon cases of primary subcutaneous echinococcoses, one AE affecting the face in the forehead area, and one CE located in the abdominal wall. To the best of our knowledge, the facial localization of AE has never been reported before. In both cases, the diagnosis had not been considered before a surgical exploration was performed. No specific precautions were taken to prevent parasitic dissemination and recurrence. Indeed, in CE, pre-operative albendazole (ABZ) administration is recommended, associated with the use of a protoscolicidal agent to protect the operative field from parasitic spillage and to avoid opening the cyst outside a controlled setting [4,5]. In AE, surgery is indicated if complete resection of the parasitic tissue is possible, followed by ABZ treatment for two years [4]. There is currently no precise recommendation for the introduction of ABZ pre-operatively. However, most expert centres currently initiate it for a period of one to six months before the operation.

**Fig 1:**
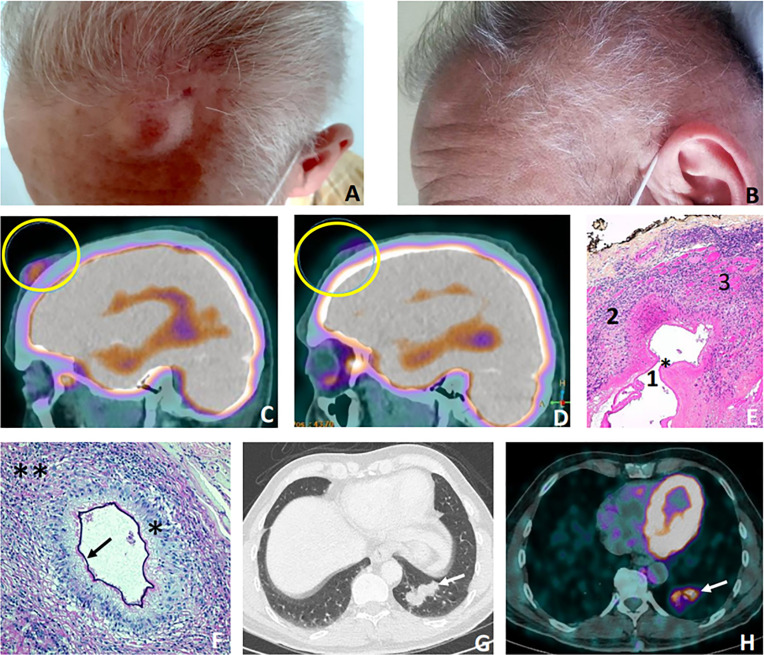
(A-H). Case 1: primary subcutaneous facial alveolar echinococcosis. **(A)** Left frontal swelling at diagnosis. **(B)** Marked regression of the lesion after 6 months of albendazole therapy. **(C-D)** FDG-PET scan sagittal images before the initiation of albendazole **(C)** and after 6 months **(D)**, with a significant decrease of the peri-metabolic activity (yellow circles). **(E-F)**. Histopathology of the frontal lesion. **(E)**. *Echinococcus (E) multilocularis* microcyst (*) delineated by the laminated layer (1), surrounded by an intense granulomatous reaction (2) and the invaded striated muscle (3). Hematoxylin-eosin stain, X10. **(F)**. A typical aspect of *E. multilocularis* lesion with a microcyst delineated by the laminated layer revealed by the periodic acid-Schiff (black arrow) and surrounded by an intense granulomatous reaction composed of macrophages with epithelioid arrangement (*) and a dense lymphocytic infiltrate (**), X20. **(G-H)**. Thoracic imagings of the alveolar echinococcosis (AE) pulmonary location. **(G)** Axial CT scan image revealing a mass in the lower lobe of the left lung (white arrow). Transparietal puncture revealed another localization of AE. **(H)** FDG-PET scan axial image showing the hypermetabolism of this lung lesion (white arrow). *E. multilocularis* PCR was positive for both skin and lung lesions.

**Case 1**. A 53-year-old man living in a rural region of eastern France, endemic for AE, consulted for a painful, non-inflammatory frontal lesion which had grown rapidly over a two-week period (Fig 1A). He had a past-history of multiple surgically-treated lipomas, and the hypothesis of a frontal lipoma was clinically considered. That is why the decision was made to perform a surgical excision without prior ultrasound examination of the lesion. The first pathological examination described a central necrobiosis and palisading histiocytes, compatible with a rheumatoid nodule. Post-operatively, the lesion increased again rapidly in size, thus requiring a second pathological opinion. It was in favor of an echinococcal infection due to the presence of microcystic structures delineated by a laminated layer revealed by the periodic acid-Schiff (PAS) stain. These microcysts infiltrated the muscular layer and were surrounded by a granulomatous inflammatory reaction (Fig 1E–1F**).** The French reference center for echinococcoses was then asked for expert advice. Indirect hemagglutination (IHA, Fumouze, antigen: *Echinococcus granulosus*) and *E.multilocularis* ELISA (Em2+ and Em18, Bordier Affinity Products) were positive (IHA titer 320, Em2 + index 2.97; Em18 index >8.00). Immunoblotting (LDBioDiagnostic) showed *E.multilocularis* specific band pattern. Moreover, pan-*Echinococcus* tissue PCR, performed according to the protocol described by Roelfsema et al. [6], and the *E. multilocularis*-specific PCR targeting the 12S rRNA gene, following the protocol described by Georges et al. [7], were both positive. A multidisciplinary decision for medical management was taken, and albendazole (ABZ) was initiated at a dosage of 200 mg twice daily.

In addition, to search for other AE localizations, a thoraco-abdomino-pelvic computed tomographic (CT) scan, a brain and abdominal magnetic resonance imaging (MRI) and a positron emission tomography with ¹^8^F-fluoro-2-deoxy-D-glucose (FDG-PET) were performed [3]. There was no primary lesion in the liver, nor in the brain; but, in addition to the peri-lesional hypermetabolism at the frontal site (Fig 1C), a solid hypermetabolic mass was found in the lower lobe of the left lung (Fig 1G–1H). To rule out neoplasia, a trans-parietal puncture was performed. The pathological examination showed eosinophil-rich necrotic material and PAS positive, acellular structures corresponding to laminated layers. The tissular *E. multilocularis* specific PCR was positive proving the extra-hepatic location of AE in the lung.

After six months on ABZ, and optimal ABZ-sulfoxide (ABZ-SOX) plasma levels (between 1.00 and 3.00 µmol/L, 4 hours after intake), the frontal AE lesion had markedly decreased in size. FDG-PET re-evaluation revealed a partial reduction in the hypermetabolism of the frontal (Fig 1D) and pulmonary lesions (respectively, SUVmax at 3.67 *vs.* 5.06 and 2.30 *vs*. 3.27, 60 minutes after injection). After 14 months on ABZ, the frontal lesion had disappeared on inspection (Fig 1B) and FDG-PET evaluation indicated a disappearance of peri-lesional activity associated with a significant decrease in the metabolic activity of the lung lesion. After 33 months on ABZ, neither the frontal lesion nor the pulmonary lesion showed any peri-lesional activity at the last FDG-PET evaluation. Finally, the serological follow-up also indicated a favorable evolution with a regular decrease of specific antibodies Em18 index: at the last evaluation, after 42 months on ABZ, Em18 index was 1.03, close to the negativity threshold (<0.90).

**Fig 2:**
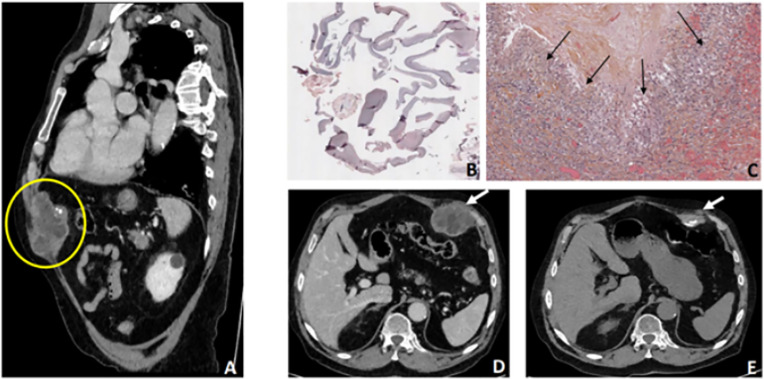
(A-E). Case 2: primary subcutaneous cystic echinococcosis located in the left subcostal area. The sub-cutaneous swelling had been present for several months and had recently grown and become painful. **(A)** Thoraco-abdomino-pelvic CT scan, which was the only imaging exam performed prior to surgery: oblique sagittal image showing a subcutaneous heterogeneous, poly-lobulated tissular lesion (yellow circle) with central hypodense portions of rounded morphology and some parietal macrocalcifications. Possible infiltration of the intraperitoneal fat in contact with the thoracoabdominal diaphragm muscle which was suspected of being invaded in its ventral portion. Following this CT scan, the diagnosis of sarcoma was considered, leading to surgical intervention. The intraoperative findings indicated that it was a cystic echinococcosis lesion **(B-C)** Histopathology of the sub-costal mass. **(B)** Hematoxylin-eosin stain of the tissue lining the cavity showing an intense granulomatous reaction (black arrows), X9.1. *E.granulosus* PCR was positive while *E.multilocularis* PCR was negative. The perilesional inflammatory reaction could be explained by a probable cystic fissuration leading to the patient’s recent symptomatology. **(C)** Hematoxylin-eosin stain showing acellular elements corresponding to the laminated layer of parasitic cyst, X0.4. **(D-E)** CT scan evolution after sub-total cystectomy followed by albendazole therapy. **(D)** Axial image showing the lesion before surgery (white arrow). **(E)** Axial image of the lesion 3 years later, the patient was still being treated with albendazole. Marked regression and increased calcifications of the cystic echinococcosis lesion (white arrow).

**Case 2**. An 81-year-old man, born in Morocco and having lived in an AE endemic region of France for more than 40 years, consulted because of a left subcostal swelling. This swelling had begun a few months earlier and had recently become larger and painful. Clinically, the patient’s general condition had not deteriorated: no weight loss, no fever, and only a moderate inflammatory syndrome (C-reactive protein at 20 mg/L, Normal range: < 5 mg/L). Initially, only one thoracic-abdominal-pelvic CT scan was performed. It showed a mixed solid and cystic lesion measuring 66x50x70 mm with an irregular wall containing arciform calcifications and a peripheral enhancement after injection of a contrast medium. This lesion developed at the left anterior abdominal muscle wall extending into the left subcostal region towards the abdominal fat and seemed to infiltrate the anterior part of the left diaphragm (Fig 2A **and** 2D**).** No additional lesions were detected and no other imaging exams were performed.

The first hypothesis was a sarcoma with a necrotic part. For this reason, a surgical exploration under local anesthesia was performed. That is when the surgeon discovered a cavity of about 4 cm containing multiple small cysts which led him to suspect echinococcosis intraoperatively. A partial cystectomy followed; multiple cysts were removed without puncture and with no injection of a protoscolecidal agent. Part of the peri-cystic area was excised after cleaning with formol. No anaphylactic reaction occurred during the procedure.

The pathological examination described a cystic cavity with acellular elements corresponding to a laminated layer (Fig 2B) with no identifiable protoscoleces or germinal layer. A thick granulomatous reaction surrounded the cavity (Fig 2C). The French reference center for echinococcoses was asked for diagnosis and expert therapeutic advice. Tissular pan-*Echinococcus* PCR was performed according to the protocol described by Roelfsema et al. [6]. *E. granulosus* (G1/G2/G3) 12S rRNA specific PCR were positive, following protocol described by Stefanić et al. [8], but the *E. multilocularis* specific PCR, (protocol described by Georges et al. [7]) was negative. Echinococcosis serologies were positive: IHA (Fumouze, antigen: *E. granulosus)*; *E. granulosus* ELISA (Bordier Affinity Product) and immunoblotting (LDBio Diagnostics), showing *E.granulosus* banding pattern. All these elements led to the diagnosis of primary sub-cutaneous CE. The atypical imaging patterns (irregular wall and peri-lesional inflammatory reaction) could be explained by a probable cystic fissuration leading to the patient’s recent symptomatology. This inflammatory reaction could also account for the CT scan appearance of perilesional pseudo-infiltration.

Considering the patient’s age and the need for major surgery to completely remove the lesion, a multidisciplinary decision for medical management was taken, and ABZ was initiated at a dosage of 200 mg twice daily, according to our expert center’s protocol, to avoid direct toxicity. Although a dosage of 400 mg twice daily is usually recommended in CE [4,5], in our expert center we prefer to initiate at a lower dosage with regular pharmacological therapeutic follow-ups, the first one from the third week of treatment, allowing us to make any necessary dosage adjustments. This dosage was maintained over the long term due to optimal ABZ-SOX plasma levels. During the post-operative follow-up, a cutaneous fistulization and two episodes of skin superinfection without microbiological isolation occurred, requiring antibiotic treatment. Six months after ABZ initiation, the lesion had decreased in size on follow-up CT scan (53x23 mm) but the fistula remained. Clinical and radiological assessment after 24 months on ABZ therapy confirmed the favorable evolution: the fistula closed and MRI indicated further reduction in lesion size (48x12 mm). The cyst appeared hypointense on both T1- and T2-weighted sequences, with no visible daughter cysts, suggesting inactivity. At the last CT-scan evaluation, after 36 months of ABZ therapy, the lesion was even smaller with increased calcifications suggesting a favorable outcome (Fig 2E).

## Methods

### Ethics statement

The two case reports described here are included in the National Reference Centre for Echinococcosis database, which complies with the European General Data Protection Regulation (GDPR) and French regulations (CNIL declaration no. 903306 v1). Both patients have provided written consent for the use of their medical data.

### Litterature review

A systematic literature search for cases of primary extrahepatic subcutaneous soft tissue echinococcosis was performed in December 2024. This review is reported in accordance with the Preferred Reporting Items for Systematic Reviews and Meta-Analyses (PRISMA) guideline. The literature search was done in Medline (National Library of Medicine, Bethesda, Maryland, USA) with no date restriction. The following search strings were used: "Echinococcosis" AND "subcutaneous" ; "Echinococcosis" AND "muscular".

A primary extra-hepatic echinococcosis lesion was defined by the absence of any current or past liver involvement (since the liver is typically the first organ affected due to portal venous dissemination following ingestion of parasite eggs) and by the absence of direct extension to neighboring organs. The presence of distant lesions was accepted, as these were considered to result from simultaneous hematogenous spread. We then selected articles based on inclusion criteria (echinococcosis of subcutaneous soft tissue; studies in English or in French; case reports, case series or reviews) and exclusion criteria (definition of a primary extra-hepatic lesion not consistent with the above-mentioned definition; abdominal imaging to search for a primary hepatic location not performed or not reported; involvement of deep muscles such as the psoas and diaphragm because they do not correspond to subcutaneous soft tissues).

Data extracted from case reports included demographics (age, sex, country), clinical characteristics (echinococcosis history, lesion site, size, duration, suspected infection route including possible inoculation), imaging features, serological and molecular test results, treatments, and outcomes.

## Results

**Fig 3:**
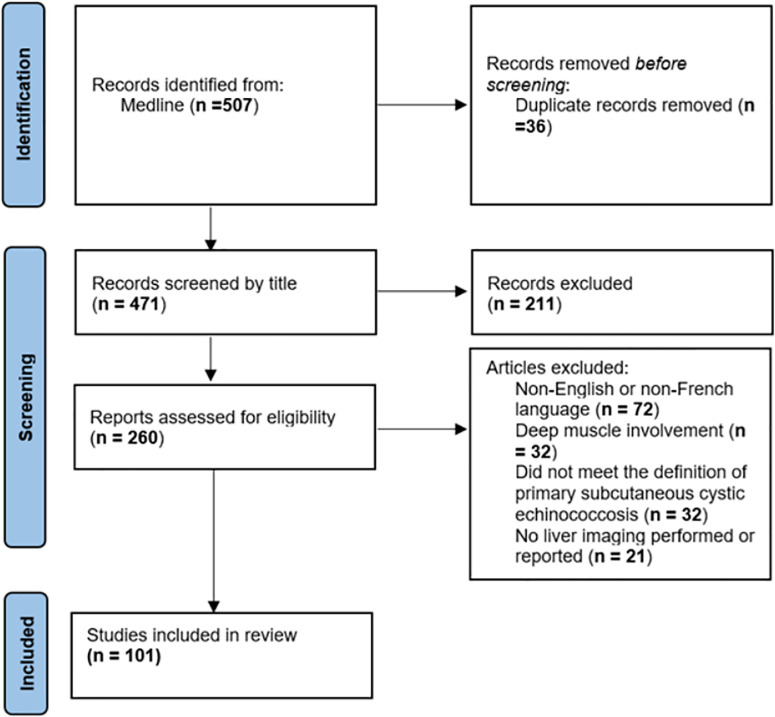
Description of the selection process for articles included in the review in accordance with the PRISMA method. PRISMA: Preferred Reporting Items for Systematic Reviews and Meta-Analyses.

Apart from case 1 presented here, no other cases of primary extrahepatic subcutaneous AE were identified. Regarding cystic echinococcosis, 102 publications (including our case) met the inclusion/exclusion criteria. (90 case reports and 12 case series) (Fig 3). S1 Table presents the characteristics of the 119 patients whose diagnosis of primary cystic echinococcosis of the subcutaneous soft tissues was retained. The majority of these reports were described in Turkey (n = 37; 36.3%) and Europe (n = 27; 26.4%).

In line with this review, 61 reported cases were women (51.3%). The median age was 40 years (IQR, 27–60). The majority of the subcutaneous cystic lesions were located in the lower limbs (n = 64; 53.8%), particularly proximally (thigh or gluteal region; n = 54; 45.4%). The other cystic locations were in the upper limb (n = 19; 16.0%), cervico-facial (n = 11; 9.2%), chest wall (n = 9; 7.6%), paraspinal (n = 8; 6.7%) and abdominal wall (n = 8; 6.7%). Cysts were described as multiple in six cases (5.0%), and an initial complication was reported in seven patients (3 vascular or nerve compressions, 2 ruptures, and 2 skin fistulisations; 5.9%). A percutaneous inoculation mechanism was suggested for three patients (2.5%): 1 post-traumatic case, 1 stab wound and 1 insect sting. None of the patients had any other echinococcosis lesion at a distant site.

Radiographic exploration was reported in 108 cases (90.8%), mainly by ultra-sonography (n = 69; 63.9%) and MRI (n = 60; 55.6%). CT-scan was reported in 31 cases (28,7%). The majority of cysts on imaging were described as multiloculated (n = 61/95; 64.2%), 34 were therefore uniloculated, including seven with a detached intra-cystic membrane (7.4%). Peripheral enhancement after contrast injection (on MRI or CT scan) was described in 17 patients, only 3 (17.6%) of whom had a lesion described as initially complicated.

The use of at least one serological tool was mentioned in 82 patients (68.9%), and the technique used was specified in 65 patients (54.6%). When reported, serological test(s) were positive in 46 patients (56.1%). Serological techniques were combined in 18 of the reported cases (27.7%), the most commonly used techniques being IHA (40/65; 61.5%) and ELISA (29/65; 44.6%). The use of Western blot was specified in eight patients (12.3%). The use of molecular biology tools was rarely reported (2/119; 1.7%).

After initiation of a therapeutic strategy, follow-up was reported in 85 patients (71.4%). The median duration of follow-up was 12 months (IQR, 10–24). Most of these cases underwent surgery (n = 83; 97.6%) and received treatment with benzimidazoles (n = 68; 80.0%). Percutaneous treatment was described in one patient (1.2%). Regarding outcome, 79 patients had no residual cystic lesions (92.9%). The size of the cyst(s) improved for two patients (1 had medical treatment alone, 1 had non-radical surgery; 2.4%). Four patients relapsed or failed therapy (1 had medical treatment alone, 1 case was complicated by a rupture of the cyst during the operation and 2 had no obvious risk factor for failure; 4.7%).

## Discussion

Soft tissue echinococcosis accounts for about 0.5 to 5% of all types of echinococcosis described and is mainly secondary to a hepatic location [9–11]. Primary echinococcosis of subcutaneous soft tissues is rare and concerns CE of the thigh or gluteal region in most reported cases [9–11]. Moreover, to our knowledge, primary extrahepatic sub-cutaneous AE and a subcutaneous AE location in the face has never been previously reported. Colleagues from Germany have recently reported an extremely rare primary AE localized in the facial area but concerning the parotid gland [12]. It appeared as a painful swelling of the right parotid gland that persisted for one year leading to partial parotidectomy. Here too, the diagnosis had not initially been made and was only confirmed on pathological examination resulting in the introduction of ABZ and complete resection of the gland [12].

The pathophysiological mechanism of primary subcutaneous soft tissue CE and AE remains unclear. The hypothesis of a direct inoculation was considered [13–15]. Experimental data indicated that a sub-cutaneous *Echinococcus multilocularis* oncosphere inoculation in mice resulted in chronic infection [16]. However, in natural human infection, embryophores require the action of bile to release the infectious oncospheres. In the subcutaneous tissue, lactic acid could favor this crucial step but the hypothesis remains controversial [10]. In this review, the majority of patients do not describe any prior skin breach, so this hypothesis of contamination by direct inoculation remains possible but not predominant. The main hypothesis of systemic dissemination therefore remained the most probable.

Concerning AE, ten cases of cutaneous involvement were identified in our review but none met the definition of a primary subcutaneous location [17–25]. However, we thought it would be interesting to describe them briefly. The majority (n = 6) involved the abdominal wall. We have divided them into 4 categories according to their presumed pathophysiological mechanisms:

Contiguous progression from the primary liver lesion (4 cases): this was the most common pathophysiological mechanism. Subcutaneous AE was located in the abdominal wall resulting in a progression *per continuitatem* from the primary liver AE lesion due to a fistula or via the falciform ligament [17–19]. In one of these cases, the diagnosis was delayed for 5 years [17].

Contiguous progression from an extrahepatic metastatic AE location (2 cases): Keutgens A *et al.* described a severe primary extrahepatic AE involving the L4 and L5 vertebrae associated with a large infiltration of the nearby paravertebral soft tissues, in continuum with the lumbar location, due to direct contiguous invasion [20]. There were also pulmonary AE foci. Interestingly, the patient, a 75-year-old man from Belgium, also suffered from severe alcoholic cirrhosis. From our experience, the hypothesis of a hepatic filter shunt from porto-systemic anastomosis or from the lymphatic circulation could be raised to explain the extra-hepatic hematogenous spreading. In fact, our group also reported a case of primary vertebral AE in a patient with documented portal hypertension related to alcoholic cirrhosis [26]. More recently, Schmidt-Hellereau K *et al.* reported a similar observation in a 79-year-old man from Germany [21]. He initially underwent surgery for a presumed subcutaneous lipoma in his upper back. Recurrent infection and fistula formation occurred during the following 2 years leading to complementary investigations. He was eventually diagnosed with vertebral AE (T3-T6) associated with a primary liver AE. Secondary pulmonary localizations were also found. In this case, the diagnostic delay for AE was 15 months.

Hematogenous dissemination from a primary AE liver lesion: Scheuring *et al*. reported the first case in 2003 [22]. The patient, a 55-year-old female, presented a tender nodulous induration of the right femoral skin and subcutaneous tissue. There was no evidence of a fistula or involvement of the femoral bone. The histopathological examination revealed a typical AE lesion, confirmed by *E.multilocularis* PCR. There was an advanced primary AE lesion in the liver associated with bone metastases (lower thoracic spine and left third toe).

Other pathophysiological mechanisms (3 cases): two additional case reports of subcutaneous abdominal AE suggested a different and particularly unusual mechanism. A 29-year-old man presented two external recurrent abdominal wall fistula and abscesses unrelated to the abdominal cavity. Histopathological examination revealed typical lesions of AE. Complementary investigations allowed the discovery of a primary liver AE lesion. As the patient mentioned that he had suffered blunt abdominal trauma 3 years before, the skin infection could have been caused by metacestode seeding from the primary liver lesion to the contiguous abdominal wall [23]. The other case concerned a 58-year-old Japanese patient who developed a subcutaneous tumor on the right side of the abdomen, thirteen years after a right partial hepatectomy for AE [24]. In the third case, the alveolar echinococcosis lesion of the chest wall appeared at the site of a dormouse bite, and 13 years passed between the onset of cutaneous symptoms and the diagnosis of the *Echinococcus multilocularis* infection, involving both hepatic and subcutaneous locations. The authors hypothesized a direct inoculation via the bite, but a systemic dissemination with preferential implantation at the site of trauma due to local inflammation appears to be a more biologically plausible explanation [25].

The differential diagnoses of superficial soft tissue masses include abscesses, sebaceous cysts, lipomas, tuberculosis, hernias, sarcomas, mycetomas, synovial cysts, necrotic soft tissue tumors, and other parasitic nodules (e.g., onchocerciasis, dirofilariasis). [10]. Primary subcutaneous AE or CE should be considered as a differential diagnosis, particularly for patients who live or have lived in endemic regions. In the evaluation of CE, ultrasound is the first-line imaging modality, as it enables detailed assessment of cyst contents, provides insight into cyst activity, and guides clinical management [4,5,27,28]. MRI is a valuable alternative, particularly due to its operator independence, allowing images review by expert centers when needed, and to its ability to assess lesions too deeply located for allowing adequate ultrasound visualization [27]. CT scan, which is less effective for analyzing cyst contents [27,28], has a more limited role, mainly contributing to the classification of cystic echinococcosis in cases of heavily calcified cysts, such as CE5 [26]. This is well illustrated by case 2, for which only a CT scan was performed. The diagnosis of CE had not been made in the absence of other imaging modalities. MRIs were performed only as part of the post-operative follow-up. For subcutaneous soft tissue CE, the appearance is similar to that of liver lesions, with multiloculated lesions (equivalent to CE2 or CE3b) or uniloculated lesions (equivalent to CE1), sometimes with membrane detachment (equivalent to CE3a). However, peripheral enhancement after injection of contrast medium, which is sometimes reported even in the absence of complications, is rarely reported in liver lesions and may give rise to doubt. In AE, MRI is the imaging modality of choice, as it allows for the identification of hyperintense T2-weighted microcysts, which are highly suggestive of this parasitic disease [4,28]. Diagnosis is more challenging with ultrasound, which typically reveals an infiltrative and heterogeneous echogenic mass with irregular borders, central necrotic areas, and possible scattered calcifications. CT scan may show an infiltrative lesion with microcalcifications and no enhancement following contrast administration [4,28]. Although serological tests have lower sensitivity in extra-hepatic involvement, their use remains valuable to support a clinical and/or radiological presumption [4]. A serological test was performed *a posteriori* in both of our cases and was highly positive. In this review, the positivity of serological tests was inconsistent and the serological techniques used were highly variable. A Western blot was rarely used, even though it is the most sensitive test [29]. In accordance with the literature on the subject, ELISA and hemagglutination should be associated with Western blot because of its high sensitivity [30]. Molecular biology on fine needle aspiration biopsy represents another useful tool to obtain a diagnosis before therapeutic decision-making [2,3,31,32]. It was particularly helpful in case 2 to distinguish AE from CE as this patient had previously lived in Morocco (a highly endemic country for CE) and then in an endemic area for AE. Only when cystic echinococcosis is suspected, such as in the pre-surgical setting to prevent dissemination, should this procedure be performed under albendazole prophylaxis.

Establishing the pre-surgical diagnosis of echinococcosis is crucial for unusual locations in order to choose the best treatment. Complete surgical resection in cases of active lesions is the best-documented treatment. [4,5,9–11,33]. Surgery should be performed under ABZ prophylaxis and, for CE, a protoscolecidal agent should be used to protect the operative area from a spillage of cystic fluid that may contain protoscoleces [4,5,34,35]. In this review, subcutaneous soft tissue CE appears to have a favourable prognosis, with only 4 authors reporting failure or relapse. However, these prognostic data are probably underestimated, as the median length of follow-up is too short to rule out late relapses, and there may be under-reporting of treatment failures. In our series of 2 cases, medical treatment alone was chosen, as in other case descriptions. [17,21,33]. In fact, ABZ-SOX has excellent diffusion in the subcutaneous tissue [17]. For CE, if surgery is not an option, medical treatment alone or even percutaneous treatment, may be considered, but only as a second line treatment in the absence of reliable data on their efficacy [36]. A multidisciplinary approach is recommended to evaluate all therapeutic options, ideally within a specialized expert center. Data from the literature about the therapeutic management of subcutaneous AE is even more limited. The infiltrative nature of AE lesions may lead to initially favoring a medical option with benzimidazole therapy. This could help avoid the risks of surgery, which can be debilitating, have a significant functional impact and is not always curative. It is the option we chose for case 1, with excellent results. The effectiveness of long-term ABZ therapy with regression of skin AE lesions has already been reported in two cases [17,21]. If surgery is considered, it is very important to carry out a thorough radiological study to precisely determine the extent of involvement, particularly in the deep planes.

Primary extrahepatic localizations of CE and AE are uncommon and probably underdiagnosed or mis-diagnosed [37–39]. In France, data from the national registry for AE, FrancEchino, indicated a prevalence of 2% for primary extrahepatic AE [40], and a more recent German series reported a prevalence of 1.3% [41]. In 2016, at the request of the Agence Santé Publique France, the French Observatory of CE was launched by the National Reference Center for Echinococcosis (NRC-E). Most of the 103 patients included between 2016 and 2023 were migrants from the mediterranean basin. Fourteen percent presented a primary extra-hepatic CE located in an organ other than the lung [42]. Unlike the data reported for AE, the proportion of primary extrahepatic forms which has been previously documented [43–45], is relatively higher and highlights the need to raise clinicians’ awareness of these atypical forms of CE.

This literature review has certain limitations. In the literature search strategy, only the term « Echinococcosis » was used; synonyms such as “hydatid“ and “alveococcosis” were not included, which may have led to an incomplete retrieval of relevant cases. Furthermore, the use of Medline as the sole database and the language restrictions applied may also have contributed to a non-exhaustive literature search. The definition of primary extrahepatic echinococcosis is not standardized, particularly with regard to whether distant (non-hepatic) lesions should be included or excluded [9,10]. If such lesions are not taken into account, case 1 would not meet the definition. Although the absence of longitudinal follow-up prevents determining which lesion appeared first, these lesions likely share the same pathophysiological mechanism of systemic dissemination. In the literature review, no cases with distant extrahepatic involvement were identified. This scenario may be rare in cystic echinococcosis, or such cases may not have been captured due to the limitations of the search strategy.

## Conclusion

Although hepatic involvement is the most frequent location in AE and CE, atypical localizations can also be observed as shown in these two cases of primary subcutaneous AE and CE. A pre-surgical diagnosis must be obtained to choose the best therapeutic option and apply recommended measures to avoid spillage, recurrence and complications. Whether for AE or CE, diagnosis relies primarily on imaging, with MRI and ultrasound being the most effective modalities respectively. Serology can be a useful diagnostic tool. In the absence of diagnostic confirmation by non-invasive methods, guided biopsy for histopathological examination and molecular analysis may be beneficial. It must be performed under albendazole therapy. Thus, both echinococcoses should be kept in mind for the differential diagnosis of soft tissue lesions in any part of the body.

## Supporting information

S1 Table Description of primary extra-hepatic subcutaneous soft tissue cystic echinococcosis: 118 cases reported in the literature from 101 publications and the case described (case 2). (PDF)
