# Supplementary material for: Unexplained subcutaneous swelling: Keep echinococcosis in mind! Report of two primary extrahepatic subcutaneous echinococcosis cases and literature review
Source: PLoS Negl Trop Dis. 2025 Sep 15;19(9):e0013511. doi: 10.1371/journal.pntd.0013511 (PMC12456795; doi:10.1371/journal.pntd.0013511)
Supplement: S1 Table — (PDF) [file pntd.0013511.s001.pdf]

S1 Table: Description of primary extra-hepatic subcutaneous soft tissue cystic echinococcosis: 118 cases reported in the literature from 101 publications and the case described (case 2).

Ref	Year	Age	Sex	History of CE	Country	Susp. Inoc.	Location	Develop . time	Cize of cyst(s) (cm)	Imag. tech.	cyst morph.	Complic.	Cont. Enh.	Serology	Mol. biology	Med.tt	Surg.tt	Per cut.tt	Follow-up time (mos)	Outcome
[1]	1993	47	F	No	Turkey	No	Thigh	2 yrs	20	CT	Multi.	Vasc. compression	No	POS (IHA)	ND	Yes	Yes	No	ND	ND
[2]	1997	50	F	No	Greece	No	Thigh	ND	5	ND	ND	No	No	ND	ND	No	Yes	No	ND	ND
[3]	1999	45	M	Yes	Spain	No	Paralumbar	5 yrs	3	CT/MRI	Multi.	No	No	POS (IHA)	ND	No	Yes	No	72	No residual lesion
[4]	2000	31	F	No	Turkey	No	Arm	4 mos	4,5/ND	MRI	Uni. + mb. detachment	No	Yes	ND	ND	No	Yes	No	ND	ND
[5]	2000	12	F	No	Turkey	No	Sub mandibular	ND	4	ND	ND	No	No	ND	ND	No	Yes	No	48	No residual lesion
[6]	2000	18	M	No	Turkey	No	Forearm	1 yr	ND	MRI	Multi.	No	No	POS (IHA)	ND	No	Yes	No	12	No residual lesion
[7]	2001	56	M	Yes	Croatia	No	Cervical	ND	7	US	Multi.	No	No	POS (IHA)	ND	Yes	Yes	No	ND	ND
[8]	2001	20	M	No	Turkey	No	Malar	ND	3	US	Uni.	No	No	NEG (ELISA)	ND	No	Yes	No	ND	ND
[9]	2001	25	M	No	Turkey	No	Shoulder	2 mos	12	MRI	Multi.	No	No	POS (IHA, ELISA, IFA)	ND	No	Yes	No	ND	ND
[10]	2001	42	F	No	Turkey	No	Thigh	ND	5	ND	ND	No	No	ND	ND	Yes	Yes	No	6	No residual lesion
[11]	2002	16	F	No	Turkey	No	Thigh	6 mos	7	MRI	Uni.	No	No	POS (IHA)	ND	Yes	Yes	No	ND	ND
[12]	2002	40	F	No	Greece	No	Thigh	ND	ND	CT/US/ MRI	Multi.	No	Yes	ND	ND	No	Yes	No	12	No residual lesion
[13]	2003	43	F	No	Turkey	No	Thigh	ND	5	CT	ND	No	No	NEG (IHA, ELISA)	ND	Yes	Yes	No	ND	ND
[14]	2003	48	M	No	Turkey	No	Thigh	10 yrs	15	US/MRI	Multi.	No	No	POS (IHA)	ND	Yes	Yes	No	7	No residual lesion
[15]	2004	34	F	No	Spain	No	Knee	7 mos	11	US/MRI	Multi.	No	No	NEG (IHA, ELISA)	ND	Yes	Yes	No	24	No residual lesion
[16]	2004	23	M	No	Turkey	No	Thigh	6 mos	30	US	Multi.	No	No	ND	ND	Yes	Yes	No	18	No residual lesion
[17]	2004	20	F	No	Turkey	No	Thigh	4 mos	20	MRI	Multi.	No	No	POS (IHA)	ND	Yes	Yes	No	12	No residual lesion
[18]	2005	62	F	No	Turkey	No	Inguinal	1 yr	4	US	ND	No	No	NEG (ND)	ND	No	Yes	No	ND	ND
[19]	2005	41	M	No	Tunisia	No	Thigh	>1 yrs	16	US/MRI	Multi.	Cut. fistula	Yes	NEG (ND)	ND	No	Yes	No	ND	ND
[19]	2005	73	M	No	Tunisia	No	Thigh	5 mos	ND	US/MRI	Uni. + mb. detachment	No	No	POS (ND)	ND	No	Yes	No	ND	ND
[20]	2005	81	M	No	Spain	No	Gluteal	2 yrs	11	MRI	Multi.	No	Yes	ND	ND	Yes	Yes	No	36	No residual lesion
[21]	2005	35	F	No	Greece	No	Thigh	6 mos	14,5	CT/MRI	Multi.	No	No	ND	ND	No	Yes	No	36	No residual lesion
[22]	2006	16	M	No	Turkey	No	Arm	6 mos	6,5	US/MRI	Uni.	No	No	NEG (ND)	ND	Yes	Yes	No	24	No residual lesion
[22]	2006	36	M	No	Turkey	No	Gluteal	3 yrs	9	MRI	Multi.	No	No	ND	ND	Yes	Yes	No	24	No residual lesion
[23]	2006	8	F	No	Turkey	No	Thigh	ND	8	MRI	Multi.	No	No	POS (IHA)	ND	Yes	Yes	No	24	No residual lesion

[24]	2006	10	F	No	Turkey	No	Retro-auricular	1 yr	1	CT/US	Multi.	No	No	NEG (ELISA)	ND	Yes	Yes	No	12	No residual lesion
[25]	2006	24	M	No	India	No	Gluteal	9 mos	8	CT/US	Uni.	No	Yes	ND	ND	Yes	Yes	No	6	No residual lesion
[26]	2007	70	M	No	Tunisia	No	Abdominal wall	1 mo	6	CT/US	ND	No	No	NEG (ND)	ND	No	Yes	No	ND	ND
[27]	2007	21	F	No	Morocco	No	Gluteal	1 yr	10	US/MRI	Multi.	No	No	NEG (ND)	ND	No	Yes	No	36	No residual lesion
[28]	2007	70	F	No	Italy	No	Thigh	4 mos	22	CT	ND	No	No	ND	ND	No	Yes	No	24	No residual lesion
[29]	2007	21	F	No	Turkey	No	Paralumbar	4 mos	3	US	ND	No	No	POS (IHA)	ND	Yes	Yes	No	24	No residual lesion
[30]	2007	34	M	No	Italy	No	Thigh	1 mo	13	US/MRI	Multi.	No	No	NEG (IHA, IFA) POS (WB)	ND	Yes	Yes	No	12	No residual lesion
[31]	2007	75	F	No	Morocco	No	Cervical	1 yr	5	CT/US	Multi.	No	No	NEG (IHA)	ND	No	Yes	No	3	No residual lesion
[32]	2008	12	F	No	India	No	Shoulder	3 mos	2	ND	ND	No	No	ND	ND	No	ND	No	ND	ND
[32]	2008	20	M	No	India	No	Paralumbar	3 mos	10	ND	ND	No	No	ND	ND	No	ND	No	ND	ND
[33]	2008	57	F	No	Austria	No	Gluteal	1 yr	15	CT	Multi.	No	No	POS (ELISA)	ND	Yes	Yes	No	ND	ND
[34]	2008	37	M	No	Turkey	No	Thigh	1 yr	7	CT/MRI	Multi.	No	Yes	ND	ND	Yes	Yes	No	41	No residual lesion
[34]	2008	34	F	No	Turkey	No	Calf	5 mos	4	MRI	Uni.	No	Yes	ND	ND	Yes	Yes	No	38	No residual lesion
[34]	2008	75	F	Yes	Turkey	No	Thigh	5 mos	20	US/MRI	Multi.	No	No	ND	ND	Yes	Yes	No	32	No residual lesion
[34]	2008	63	F	Yes	Turkey	No	Thigh	8 yrs	20	MRI	Multi.	No	No	POS (Weinberg)	ND	Yes	Yes	No	24	No residual lesion
[34]	2008	33	M	No	Turkey	No	Calf	3 yrs	6	US	ND	No	No	POS (Weinberg)	ND	No	Yes	No	7	Relapse
[35]	2008	64	M	No	Turkey	No	Thigh	1 yr	9	CT/US	Multi.	No	No	NEG (IHA)	ND	Yes	Yes	No	36	No residual lesion
[35]	2008	67	M	No	Turkey	No	Hand	ND	3	ND	ND	No	No	NEG (IHA)	ND	Yes	Yes	No	36	No residual lesion
[36]	2008	73	M	No	Greece	No	Gluteal	8 yrs	6	US	Uni.	No	No	POS (ELISA, WB)	ND	Yes	Yes	No	36	No residual lesion
[37]	2008	65	M	No	Spain	No	Knee	7 yrs	6	CT/MRI	Multi.	No	No	NEG (ND)	ND	Yes	Yes	No	36	No residual lesion
[38]	2008	14	M	No	India	No	Abdominal wall	3 mos	11	US	Multi.	No	No	POS (ELISA)	ND	Yes	Yes	No	24	No residual lesion
[39]	2008	47	F	No	Turkey	No	Arm	3 mos	13	MRI	Multi.	No	No	NEG (IHA)	ND	Yes	Yes	No	18	No residual lesion
[40]	2008	29	F	No	Turkey	No	Thigh	ND	5	US/MRI	Multi.	No	No	POS (IHA, WB)	ND	Yes	Yes	No	12	No residual lesion
[41]	2008	37	F	No	Turkey	No	Thigh	3 mos	16	MRI	Multi.	No	No	POS (IHA)	ND	Yes	Yes	No	12	No residual lesion
[41]	2008	17	F	No	Turkey	No	Shoulder	6 mos	11	MRI	Multi.	No	No	POS (IHA)	ND	Yes	Yes	No	12	No residual lesion
[42]	2009	20	M	No	Turkey	No	Thigh	7 mos	17	US/MRI	Multi.	No	No	POS (ELISA)	ND	Yes	Yes	No	ND	ND
[43]	2009	40	F	No	Morocco	No	Forearm	5 yrs	12	CT/US	Multi.	Nerve compression	No	NEG (ND)	ND	Yes	Yes	No	24	No residual lesion

[44]	2009	72	M	No	Greece	No	Gluteal	6 mos	8	CT	Uni.	No	No	NEG (ELISA)	ND	No	Yes	No	12	No residual lesion
[45]	2009	28	M	Yes	India	No	Chest wall	7 yrs	10	ND	ND	No	No	ND	ND	Yes	Yes	No	12	No residual lesion
[46]	2010	84	F	No	Turkey	No	Thigh	ND	8/5	US/MRI	Multi.	Cut. fistula	No	ND	ND	Yes	No	No	ND	ND
[47]	2010	40	F	No	India	No	Paralumbar	4 mos	20	CT/US	Multi.	No	No	POS (ELISA)	ND	No	Yes	No	ND	ND
[48]	2010	25	F	No	Tunisia	No	Thigh	ND	8	MRI	Uni. + mb. detachment	No	No	POS (ELISA)	ND	No	Yes	No	36	No residual lesion
[49]	2011	21	F	No	Turkey	No	Cervical	3 mos	5	US/MRI	Uni.	No	Yes	POS (IHA)	ND	Yes	Yes	No	ND	ND
[50]	2011	27	M	No	Iran	No	Axillary	1,5 yrs	10	US/MRI	Multi.	No	No	ND	ND	Yes	Yes	No	ND	ND
[51]	2011	42	M	No	Tunisia	No	Paralumbar	4 yrs	8	CT/US	Uni.	No	No	NEG (ELISA)	ND	No	Yes	No	72	No residual lesion
[52]	2011	70	M	No	Morocco	No	Abdominal wall	6 mos	6	US	Uni.	No	No	NEG (ND)	ND	No	Yes	No	24	No residual lesion
[53]	2011	45	M	No	India	No	Temporal	2 yrs	6,2	CT	Uni.	No	No	ND	ND	Yes	Yes	No	22	No residual lesion
[54]	2011	30	F	No	India	No	Thigh	4 yrs	12	MRI	Multi.	No	No	ND	ND	Yes	Yes	No	12	No residual lesion
[55]	2011	45	F	No	Turkey	No	Axillary	2 mos	3	US	Uni.	No	No	NEG (IHA, ELISA)	ND	Yes	Yes	No	12	No residual lesion
[56]	2011	63	M	No	Hungary	Yes (Insect sting)	Knee	ND	4	US	Multi.	No	No	ND	ND	Yes	Yes	No	10	Relapse
[57]	2012	18	M	No	Albania	No	Thigh	3 mos	10	US/MRI	Uni. + mb. detachment	Nerve compression	No	NEG (IHA, ELISA)	ND	No	Yes	No	30	No residual lesion
[58]	2012	14	F	No	Morocco	No	Thigh	2 yrs	7	US/MRI	Uni.	No	Yes	NEG (IHA, ELISA)	ND	No	Yes	No	24	No residual lesion
[59]	2012	33	F	No	Iran	No	Axillary	4 mos	5	US	Uni.	No	No	NEG (ELISA)	ND	Yes	Yes	No	12	No residual lesion
[60]	2012	38	M	No	India	No	Thigh	10 mos	7	CT/US	Multi.	No	No	ND	ND	Yes	Yes	No	8	No residual lesion
[61]	2012	60	F	No	India	No	Abdominal wall	1 yr	15	CT/US	Multi.	No	No	ND	ND	Yes	Yes	No	6	No residual lesion
[62]	2012	21	M	No	India	No	Arm	ND	12	US/MRI	Uni. + mb. detachment	No	No	ND	ND	No	Yes	No	6	No residual lesion
[63]	2012	53	F	No	Tunisia	No	Cervical	3 mos	14	CT	Multi.	No	No	NEG (IHA, ELISA)	ND	Yes	Yes	No	4	No residual lesion
[64]	2013	53	M	No	Saudi Arabia	No	Thigh	10 yrs	13,7	MRI	Multi.	No	No	POS (ND)	ND	No	Yes	No	ND	ND
[65]	2013	48	M	No	Turkey	No	Shoulder	8 mos	10	MRI	Multi.	No	No	POS (IHA)	ND	Yes	Yes	No	ND	ND
[66]	2013	63	M	No	Turkey	No	Abdominal wall	1 yr	5	US	Multi.	No	No	NEG (ELISA)	ND	Yes	Yes	No	ND	ND
[67]	2013	60	F	No	France	No	Thigh	6 mos	10	US	Uni.	No	No	POS (ELISA, WB)	ND	Yes	Yes	No	30	Relapse
[68]	2013	30	F	No	Turkey	No	Thigh	3 mos	28	US/MRI	Uni. + mb. detachment	Infection or rupture	Yes	ND	ND	Yes	Yes	No	12	No residual lesion
[69]	2013	53	F	No	Turkey	No	Pre-auricular	1 week	4	US	ND	No	No	NEG (IgE)	ND	Yes	Yes	No	6	No residual lesion
[69]	2013	37	F	No	Turkey	No	Scapular	1 mo	10	US	ND	No	No	NEG (IgE)	ND	Yes	Yes	No	6	No residual lesion

[70]	2013	68	M	No	Italy	Yes (trauma)	Shoulder	6 mos	10	CT/MRI	Multi.	No	Yes	ND	ND	Yes	Yes	No	6	No residual lesion
[71]	2013	44	F	No	Turkey	No	Thigh	1 yr	20	CT/US/ MRI	Uni.	No	Yes	POS (IHA)	ND	Yes	No	Yes	6	Improvement
[72]	2014	64	F	No	Turkey	No	Abdominal wall	4 yrs	4	ND	ND	No	No	ND	ND	No	Yes	No	ND	ND
[73]	2014	70	F	No	India	No	Thigh	2 yrs	10 /4,5	US	Multi.	No	No	ND	ND	No	Yes	No	ND	ND
[74]	2014	7	M	No	Turkey	No	Subclavicular	1 yr	4	CT/US	Uni.	No	No	NEG (IHA, IgE)	ND	No	Yes	No	ND	ND
[75]	2014	70	M	No	Turkey	No	Arm	9 mos	10/5	US/MRI	Multi.	No	No	NEG (IHA)	ND	Yes	Yes	No	12	No residual lesion
[76]	2014	62	F	No	Turkey	No	Paraspinal	5 mos	4	MRI	Uni.	No	Yes	POS (IHA)	ND	Yes	Yes	No	3	No residual lesion
[77]	2015	50	F	Yes	Morocco	No	Calf	2 yrs	7/4	CT/MRI	Uni.	No	Yes	POS (ELISA)	ND	Yes	Yes	No	24	No residual lesion
[78]	2015	65	M	No	France	No	Paralumbar	30 yrs	25	US/MRI	Multi.	No	No	POS (ELISA, WB)	ND	Yes	Yes	No	12	No residual lesion
[79]	2015	57	M	No	Greece	Yes (Stab wound)	Pectoral	7 yrs	11	CT	Multi.	No	No	POS (ND)	ND	Yes	Yes	No	6	No residual lesion
[80]	2016	33	M	No	France	No	Thigh	ND	9,4	MRI	Multi.	No	No	POS (ELISA, WB)	ND	Yes	Yes	No	4	No residual lesion
[81]	2016	36	F	No	Turkey	No	Thigh	9 mos	7	US/MRI	Uni. + mb. detachment	No	No	POS (IHA)	ND	Yes	Yes	No	ND	ND
[82]	2016	62	F	No	India	No	Thigh	7 yrs	8	US	Multi.	No	No	NEG (ND)	ND	Yes	Yes	No	24	No residual lesion
[83]	2016	17	F	No	Morocco	No	Arm	2 mos	1,5	ND	ND	No	No	NEG (ND)	ND	Yes	Yes	No	6	No residual lesion
[84]	2016	34	M	No	Iran	No	Calf	5 mos	8	US	ND	No	No	POS (ND)	ND	Yes	Yes	No	6	No residual lesion
[85]	2017	41	M	No	Italy	No	Thigh	ND	19	US/MRI	Multi.	No	No	ND	ND	Yes	Yes	No	12	No residual lesion
[86]	2017	77	F	No	Spain	No	Thigh	1 yr	3,6/1,6	US/MRI	Uni.	No	Yes	ND	ND	Yes	Yes	No	12	No residual lesion
[87]	2018	37	M	No	Iraq	No	Thigh	3 weeks	10	US/MRI	Uni.	No	No	POS (ELISA)	ND	Yes	Yes	No	ND	ND
[88]	2018	51	M	No	Spain	No	Thigh	ND	12	US/MRI	Multi.	No	No	NEG (ELISA)	ND	No	Yes	No	24	No residual lesion
[89]	2018	21	F	No	Pakistan	No	Thigh	3 yrs	17,4	US/MRI	Multi.	No	No	ND	ND	Yes	Yes	No	11	No residual lesion
[90]	2019	52	F	No	Spain	No	Pectoral	8 mos	6	CT/MRI	Multi.	No	No	POS (ND)	ND	Yes	Yes	No	ND	ND
[91]	2019	36	M	No	Iran	No	Arm	6 mos	ND	MRI	Uni.	No	No	POS (ELISA, IgE)	ND	No	Yes	No	ND	ND
[92]	2019	37	M	No	Austria	No	Calf	1 yr	20	US	Multi.	No	No	POS (ELISA, WB)	ND	Yes	Yes	No	24	No residual lesion
[93]	2020	32	M	No	India	No	Pre-auricular	2 mos	2	ND	ND	No	No	ND	ND	No	Yes	No	ND	ND
[94]	2020	65	M	No	Canada	No	Pectoral	2 yrs	4	MRI	Multi.	No	No	NEG (ELISA)	POS	Yes	Yes	No	48	No residual lesion
[95]	2020	22	M	No	Ethiopia	No	Foot	ND	2,5	ND	ND	No	No	ND	ND	Yes	Yes	No	6	No residual lesion

[96]	2021	70	M	No	Spain	No	Thigh	2 weeks	10	US/MRI	Multi.	No	Yes	POS (IHA, IgE)	ND	Yes	No	No	10	Treatment failure
[97]	2022	55	F	No	Tunisia	No	Paralumbar	2 yrs	5,5	CT/US/ MRI	Multi.	No	No	NEG (ND)	ND	Yes	Yes	No	ND	ND
[98]	2022	33	F	No	Peru	No	Thigh	2 yrs	10	US	ND	No	No	ND	ND	Yes	Yes	No	ND	ND
[98]	2022	30	F	No	Peru	No	Thigh	18 mos	12	US	ND	No	No	ND	ND	Yes	Yes	No	ND	ND
[99]	2022	55	M	No	Iran	No	Thigh	35 yrs	27	US/MRI	Multi.	No	No	ND	ND	Yes	Yes	No	12	No residual lesion
[100]	2022	24	M	No	Turkey	No	Subclavicular	ND	6	CT	Uni.	No	No	POS (IHA)	ND	Yes	Yes	No	12	No residual lesion
[100]	2022	35	F	No	Turkey	No	Neck	ND	3	US	Uni.	No	No	NEG (IHA)	ND	Yes	Yes	No	12	No residual lesion
[100]	2022	45	F	No	Turkey	No	Chest wall	ND	11	US	Uni.	No	No	POS (IHA)	ND	Yes	Yes	No	12	No residual lesion
[100]	2022	67	M	No	Turkey	No	Gluteal	ND	10	US	ND	No	No	POS (IHA)	ND	Yes	Yes	No	12	No residual lesion
[100]	2022	48	F	No	Turkey	No	Chest wall	ND	5	US	Uni.	No	No	POS (IHA)	ND	Yes	Yes	No	12	No residual lesion
[100]	2022	21	F	No	Turkey	No	Thigh	ND	4,5	MRI	Multi.	No	No	POS (IHA)	ND	Yes	Yes	No	12	No residual lesion
[100]	2022	45	M	No	Turkey	No	Abdominal wall	ND	7	US	ND	No	No	POS (IHA)	ND	Yes	Yes	No	12	No residual lesion
[101]	2023	40	F	No	Iran	No	Arm	1 yr	6,5	US	Uni.	No	No	NEG (ND)	ND	Yes	Yes	No	6	No residual lesion
Case n°2	2025	81	M	No	France	No	Sub-costal	ND	7	CT/MRI	Multi.	Rupture	Yes	POS (IHA, ELISA, WB)	POS	Yes	Yes	No	36	Improvement

CE = cystic echinococcosis ; susp.inoc. = suspected inoculation ; develop. = development ; morph. = morphology ; complic. = complication ; cont.enh = contrast enhancement ; mol. = molecular ; med.tt = medical treatment ; sur.tt = surgical treatment ; per cut.tt = percutaneous treatment ; mo = month ; yr= year ; ND = no data ; POS = positive ; NEG = negative ; CT = computed tomography ; US = ultrasonography ; MRI = Magnetic resonance imaging ; IHA = indirect hemagglutination test ; IFA = indirect fluorescence antibody ; ID = immunodiffusion test ; ELISA = Enzyme-linked immunosorbent assay ; multi. = multiloculated ; uni. = uniloculated ; Mb = membrane.

REFERENCES:

1. Yörükoğlu Y, Zengin M, Dolgun A, Nazliel K, Salman E, Paşaoğlu E, et al. Primary muscular hydatid cyst causing arterial insufficiency: case report and literature review. Angiology. 1993;44: 399–401. doi:10.1177/000331979304400509

2. Voucharas C, Papaioannidis D, Papamichael K. Subcutaneous mass of the right thigh. Postgrad Med J. 1998;74: 287–288. doi:10.1136/pgmj.74.871.287

3. García-Alvarez F, Torcal J, Salinas JC, Güemes A, Navarro AC, Lozano R. Primary hydatid disease in lumbar muscles. Acta Orthop Belg. 1999;65: 521–524. Available from: https://www.actaorthopaedica.be/archive/volume-65/issue-4/article/primary-hydatid-disease-in-lumbar-muscles/

4. Tacal T, Altinok D, Yildiz YT, Altinok G. Coexistence of intramuscular hydatid cyst and tapeworm. AJR Am J Roentgenol. 2000;174: 575–576. doi:10.2214/ajr.174.2.1740575

5. Ok E, SÃ¶zÃ¼er EM. Solitary subcutaneous hydatid cyst: a case report. Am J Trop Med Hyg. 2000;62: 583–584. doi:10.4269/ajtmh.2000.62.583

6. Bayram M, Sirikci A. Hydatic cyst located intermuscular area of the forearm: MR imaging findings. Eur J Radiol. 2000;36: 130–132. doi:10.1016/s0720-048x(00)00188-1

7. Gluncić I, Roje Z, Bradarić N, Petricević A, Pisac VP, Gluncić V. Primary echinococcosis of the sternocleidomastoid muscle. Croat Med J. 2001;42: 196–198. Available from: https://pubmed.ncbi.nlm.nih.gov/11259745/

8. Oztürk S, Devec M, Yildirim S. Hydatid cyst in the soft tissue of the face without any primary. Ann Plast Surg. 2001;46: 170–173. doi:10.1097/00000637-200102000-00016

9. Tatari H, Baran O, Sanlidağ T, Göre O, Ak D, Manisali M, et al. Primary intramuscular hydatidosis of supraspinatus muscle. Arch Orthop Trauma Surg. 2001;121: 93–94. doi:10.1007/pl00013775

10. Acar T, Taçyildiz R, Tuncal S. Isolated hydatic cyst in the subcutaneous tissue. Turk J Med Sci. 2001;31: 575–576. Available from: https://journals.tubitak.gov.tr/medical/vol31/iss6/20/

11. Keskin D, Ezirmik N, Karsan O, Gürsan N. Primary hydatidosis of the gracilis muscle in a girl. J Int Med Res. 2002;30: 449–451. doi:10.1177/147323000203000415

12. Alexiadis G, Lambropoulou M, Deftereos S, Papadopoulos N, Manavis J. Primary muscular hydatidosis. US, CT and MR findings. Acta Radiol Stockh Swed 1987. 2002;43: 428–430. doi:10.1080/j.1600-0455.2002.430416.x

13. Orhan Z, Kara H, Tuzuner T, Sencan I, Alper M. Primary subcutaneous cyst hydatic disease in proximal thigh: an unusual localisation: a case report. BMC Musculoskelet Disord. 2003;4: 25. doi:10.1186/1471-2474-4-25

14. Ozkoç G, Akpinar S, Hersekli MA, Ozalay M, Tandoğan R. Primary hydatid disease of the quadriceps muscle: a rare localization. Arch Orthop Trauma Surg. 2003;123: 314–316. doi:10.1007/s00402-003-0512-1

15. Guiral J, Rodrigo A, Tello E. Subcutaneous echinococcosis of the knee. The Lancet. 2004;363: 38. doi:10.1016/S0140-6736(03)15168-9

16. Koc Z, Ağildere AM, Yalcin O, Pourbagher A, Pourbagher M. Primary hydatid cyst in the anterior thigh: Sonographic findings. J Clin Ultrasound JCU. 2004;32: 358–360. doi:10.1002/jcu.20044

17. Buyukbebeci O, Gulec A, Gokalp A, Karakurum G. Combined treatment of primary hydatid disease of the vastus lateralis muscle. Orthopedics. 2004;27: 621–622. doi:10.3928/0147-7447-20040601-21

18. Kiyak G, Ergül E, Korukluoglu B. Primary Hydatid Disease of the Soft Tissue. Acta Chir Belg. 2007;107: 452–453. doi:10.1080/00015458.2007.11680096

19. Alouini Mekki R, Mhiri Souei M, Allani M, Bahri M, Arifa N, Jemni Gharbi H, et al. Kyste hydatique des tissus mous : apport de l’IRM (À propos de trois observations). J Radiol. 2005;86: 421–425. doi:10.1016/S0221-0363(05)81376-9

20. Combalia A, Sastre-Solsona S. Hydatid cyst of gluteus muscle. Two cases. Review of the literature. Joint Bone Spine. 2005;72: 430–432. doi:10.1016/j.jbspin.2004.11.007

21. Kazakos CJ, Galanis VG, Verettas D a. J, Polychronidis A, Simopoulos C. Primary hydatid disease in femoral muscles. J Int Med Res. 2005;33: 703–706. doi:10.1177/147323000503300613

22. Ates M, Karakaplan M. Hydatid cyst in the biceps and gluteus muscles: case report. Surg Infect. 2007;8: 475–478. doi:10.1089/sur.2006.040

23. Duygulu F, Karaoğlu S, Erdoğan N, Yildiz O. Primary hydatid cyst of the thigh: a case report of an unusual localization. Turk J Pediatr. 2006;48: 256–259. Available from: https://turkjpediatr.org/article/view/2640#

24. Gurbuz M, Ozudogru E, Cakli H, Kabukcuoglu S, Dogan N. A retroauricular situated primary hydatid cyst: a case report. Turk Arch Otorhinolaryngol-TURK OTORINOLARENGOLOJI ARSIVI. 2006;44. Available from: https://avesis.ogu.edu.tr/yayin/eb91f1b4-efa3-4dc6-ae25-da5fcbba5247/a-retroauricular-situated-primary-hydatid-cyst-a-case-report

25. Haque F, Harris SH, Khan R, Abbas SZ. Primary hydatidosis of gluteus maximus. J Postgrad Med. 2006;52: 300–301. Available from: http://www.bioline.org.br/request?jp06101

26. Bedioui H, Makni A, Nouira K, Mekni A, Daghfous A, Ayadi S, et al. [Subcutaneous hydatid cyst. Case report of an exceptional location]. Med Trop (Mars). 2007;67: 181–182. Available from: https://pubmed.ncbi.nlm.nih.gov/17691440/

27. Daoudi A, Loudiyi W-D, Elibrahimi A, Elmrini A, Chakour K, Boutayeb F. [Solitary subcutaneous hydatid cyst of gluteal area: an unusual localisation. A case report]. Ann Chir Plast Esthet. 2008;53: 448–451. doi:10.1016/j.anplas.2007.09.002

28. Insabato L, Marino G, Fazioli F, Iacono V, Mascolo M, Palombini L. Primary intramuscular infestation of Echinococcus granulosus misdiagnosed as a soft tissue tumor: a case report. Acta Cytol. 2007;51: 631–633. doi:10.1159/000325814

29. Dogmus M, Kaplan M, Salman B, Yilmaz T. Unusual Presence of Hydatid Disease In Subcutaneous Tissue: A Case Report. New J Med. 2007;24: 246–248. Available from: https://www.researchgate.net/publication/228710562_Unusual_Presence_of_Hydatid_Disease_In_Subcutaneous_Tissue_A_Case_Report

30. Vicidomini S, Cancrini G, Gabrielli S, Naspetti R, Bartoloni A. Muscular cystic hydatidosis: case report. BMC Infect Dis. 2007;7: 23. doi:10.1186/1471-2334-7-23

31. Benhammou A, Benbouzid MA, Bencheikh R, Boulaich M, Essakali L, Kzadri M. Hydatid cyst of the neck. B-ENT. 2007;3: 201–203. Available from: https://www.b-ent.be/en/hydatid-cyst-of-the-neck-13772

32. Gupta R, Mathur SR, Agarwala S, Kaushal S, Srivastav A. Primary soft tissue hydatidosis: Aspiration cytological diagnosis in two cases. Diagn Cytopathol. 2008;36: 884–886. doi:10.1002/dc.20936

33. Steurer S, Auer H. Primary cystic echinococcosis in the subcutaneous gluteal region - a case report. Wien Klin Wochenschr. 2008;120: 101–103. doi:10.1007/s00508-008-1039-9

34. Basarir K, Saglik Y, Yildiz Y, Yetis T, Cebesoy O. Primary muscular hydatidosis mimicking soft tissue tumour: a report of five cases. J Orthop Surg Hong Kong. 2008;16: 368–372. doi:10.1177/230949900801600321

35. Dirican A, Unal B, Kayaalp C, Kirimlioglu V. Subcutaneous hydatid cysts occurring in the palm and the thigh: two case reports. J Med Case Reports. 2008;2: 273. doi:10.1186/1752-1947-2-273

36. Safioleas M, Nikiteas N, Stamatakos M, Safioleas C, Manti C h., Revenas C, et al. Echinococcal cyst of the subcutaneous tissue: A rare case report. Parasitol Int. 2008;57: 236–238. doi:10.1016/j.parint.2007.11.002

37. Seijas R, Catalán-Larracoechea JM, Ares-Rodríguez O, Joshi N, de la Fuente JPG, Pérez-Domínguez M. Primary hydatid cyst of skeletal muscle affecting the knee: a case report. Arch Orthop Trauma Surg. 2009;129: 39–41. doi:10.1007/s00402-008-0583-0

38. Srivastava P, Gangopadhyay AN, Upadhyaya VD, Sharma SP, Jaiman R. An unusual presentation of hydatid cyst in anterior abdominal wall. Kathmandu Univ Med J KUMJ. 2008;6: 511–513. doi:10.3126/kumj.v6i4.1746

39. Karapinar H, Yağdi S, Durmuş K, Sener M. Primary hydatid disease of the biceps brachii. J Shoulder Elbow Surg. 2008;17: e6-8. doi:10.1016/j.jse.2007.06.026

40. Parsak CK, Eray IC, Sakman G, Eray SI, Gumurdurlu D, Akcam T, et al. Hydatid disease involvement of primary subcutaneous tissue in the posterior proximal thigh – an unusual localization. Int J Dermatol. 2008;47: 417–418. doi:10.1111/j.1365-4632.2008.03387.x

41. Yüksel BC, Akbulut S, Hengirmen S. A minimally invasive treatment option in primary muscular hydatid cyst: report of 2 cases. Can J Surg J Can Chir. 2008;51: 153–154. Available from: https://www.canjsurg.ca/content/51/2/153.long

42. Acar A, Rodop O, Yenilmez E, Baylan O, Oncül O. Case report: primary localization of a hydatid cyst in the adductor brevis muscle. Turk Parazitolojii Derg. 2009;33: 174–176. Available from: https://pubmed.ncbi.nlm.nih.gov/19598099/

43. Daoudi A, Shimi M, Lahrach K, Elibrahimi A, Loudiyi WD, Amar MF, et al. [Isolated echinococcosis of the flexor digitorum profondus: a case report]. Chir Main. 2009;28: 175–179. doi:10.1016/j.main.2009.03.004

44. Manouras A, Lagoudianakis EE, Markogiannakis H, Larentzakis A, Kekis P, Filis K, et al. Primary hydatidosis of the gluteus muscles: report of three cases. Ir J Med Sci. 2009;178: 359–362. doi:10.1007/s11845-008-0160-0

45. Singh S, Khichy S, Singh M, Gill JS. Recurrent solitary hydatid cyst of the subcutaneous tissue. Indian J Surg. 2009;71: 162–164. doi:10.1007/s12262-009-0044-1

46. Ozkan HS, Sahin B. Primary hydatid disease of subcutaneous tissue in the leg. Clin Exp Dermatol. 2010;35: 915–916. doi:10.1111/j.1365-2230.2010.03843.x

47. Parray FQ, Ahmad SZ, Sherwani AY, Chowdri NA, Wani KA. Primary paraspinal hydatid cyst: a rare presentation of Echinococcosis. Int J Surg Lond Engl. 2010;8: 404–406. doi:10.1016/j.ijsu.2010.04.011

48. Hamdi MF, Touati B, Abid A. Primary hydatid cyst of the biceps femoris. Musculoskelet Surg. 2010;94: 59–61. doi:10.1007/s12306-010-0056-9

49. Iynen I, Sogut O, Guldur ME, Kose R, Kaya H, Bozkus F. Primary Hydatid Cyst: An Unusual Cause of a Mass in the Supraclavicular Region of the Neck. J Clin Med Res. 2011;3: 52–54. doi:10.4021/jocmr495w

50. Motie MR, Rezapanah A, Pezeshki Rad M, Razavian H, Azadmand A, Khajeh M. Primary localization of a hydatid cyst in the latissimus dorsi muscle: an unusual location. Surg Infect. 2011;12: 401–403. doi:10.1089/sur.2010.077

51. Sallami S, Ayari K, Oueslati B, Miladi M. Isolated subcutaneous hydatid cyst. Tunis Med. 2011;89: 314–315. Available from: https://latunisiemedicale.com/index.php/tunismed/article/view/1677

52. Ousadden A, Elbouhaddouti H, Ibnmajdoub KH, Mazaz K, Aittaleb K. A solitary primary subcutaneous hydatid cyst in the abdominal wall of a 70-year-old woman: a case report. J Med Case Reports. 2011;5: 270. doi:10.1186/1752-1947-5-270

53. Bansal C, Lal N, Jain RC, Srivastava AN, Fatima U. Primary hydatid cyst in the soft tissue of the face: an exceptional occurrence. Indian J Dermatol. 2011;56: 768–770. doi:10.4103/0019-5154.91852

54. Pathak TK, Roy S, Das S, Achar A, Biswas AK. Solitary hydatid cyst in thigh without any detectable primary site. JPMA J Pak Med Assoc. 2011;61: 1244–1245. Available from: https://pubmed.ncbi.nlm.nih.gov/22355979/

55. Ozsoy M, Keles C, Kahya M, Keles G. Primary echinococcal cyst in the axillary region. J Infect Dev Ctries. 2011;5: 825–827. doi:10.3855/jidc.1589

56. Battyany I, Andrea L, Nagy KK. Subcutaneous hydatid cyst in the popliteal fossa at the site of a previous wasp sting. Diagn Interv Radiol Ank Turk. 2011;17: 163–165. doi:10.4261/1305-3825.DIR.2933-09.1

57. Alimehmeti R, Seferi A, Rroji A, Alimehmeti M. Saphenous neuropathy due to large hydatid cyst within long adductor muscle: case report and literature review. J Infect Dev Ctries. 2012;6: 531–535. doi:10.3855/jidc.1766

58. Mahmoudi A, Rachidi CA, Khattala K, Chater L, Bouabdallah Y, Afifi MA. Primary subcutaneous hydatid cyst in the left distal thigh. Eur J Orthop Surg Traumatol Orthop Traumatol. 2012;22 Suppl 1: 177–180. doi:10.1007/s00590-012-1020-1

59. Zangeneh M, Amerion M, Siadat SD, Alijani M. Primary Hydatid Cyst of the Axillary Region: A Case Report. Case Rep Med. 2012;2012: 362610. doi:10.1155/2012/362610

60. Gupta A, Singal R, Gupta S, Singal R. Hydatid cyst of thigh diagnosed on ultrasonography - a rare case report. J Med Life. 2012;5: 196–197. Available from: https://pmc.ncbi.nlm.nih.gov/articles/PMC3391869/

61. Abhishek V, Patil VS, Mohan U, Shivswamy BS. Abdominal Wall Hydatid Cyst: Case Report and Review of Literature. Case Rep Surg. 2012;2012: 583294. doi:10.1155/2012/583294

62. Ghonge NP, Rajan S, Aggarwal B, Sahu AK. Imaging of ruptured endocyst in an isolated intramuscular hydatid cyst - The Scroll appearance. J Radiol Case Rep. 2012;6: 17–21. doi:10.3941/jrcr.v6i8.739

63. Jarboui S, Hlel A, Daghfous A, Bakkey MA, Sboui I. Unusual location of primary hydatid cyst: soft tissue mass in the supraclavicular region of the neck. Case Rep Med. 2012;2012: 484638. doi:10.1155/2012/484638

64. Almadani N, Almutairi B, Alassiri AH. Primary Subcutaneous Hydatid Cyst with Palisading Granulomatous Reaction. Case Rep Pathol. 2013;2013: 126541. doi:10.1155/2013/126541

65. Boyaci N, Boyaci A, Karakas E, Altay MA. Primer hydatid cyst of the deltoid muscle. BMJ Case Rep. 2013;2013: bcr2013200209. doi:10.1136/bcr-2013-200209

66. Burgazli KM, Ozdemir CS, Beken Ozdemir E, Mericliler M, Polat ZP. Unusual localization of a primary hydatid cyst: a subcutaneous mass in the paraumbilical region. Eur Rev Med Pharmacol Sci. 2013;17: 1766–1768. Available from: https://pubmed.ncbi.nlm.nih.gov/23852901/

67. Argy N, Abou Bacar A, Boeri C, Lohmann C, Pfaff AW, Hansmann Y, et al. Primary musculoskeletal hydatid cyst of the thigh: Diagnostic and curative challenge for an unusual localization. Can J Infect Dis Med Microbiol J Can Mal Infect Microbiol Medicale. 2013;24: e99–e101. doi:10.1155/2013/829471

68. Karakaş E, Çullu N, Altay N, Ozturk IA. Infected primary hydatid cyst located in the Sartorius muscle. Indian J Med Microbiol. 2013;31: 412–414. doi:10.4103/0255-0857.118878

69. Ay S, Okuş A, Demirgül R, Eryılmaz MA, Atay A. Primary subcutaneous cyst hydatid: presentation of two cases. Turk Parazitolojii Derg. 2013;37: 219–221. doi:10.5152/tpd.2013.49

70. Vecchio R, Marchese S, Ferla F, Spataro L, Intagliata E. Solitary subcutaneous hydatid cyst: review of the literature and report of a new case in the deltoid region. Parasitol Int. 2013;62: 487–493. doi:10.1016/j.parint.2013.06.013

71. Yucesoy C, Ozturk E, Hekimoglu B. Radiologic findings and percutaneous treatment of a rare giant soft tissue hydatid cyst. JBR-BTR Organe Soc R Belge Radiol SRBR Orgaan Van K Belg Ver Voor Radiol KBVR. 2013;96: 286–289. doi:10.5334/jbr-btr.403

72. Akkaya H, Akkaya B, Gönülcü S. Hydatid disease involving some rare sites in the body. Turk Parazitolojii Derg. 2015;39: 78–82. doi:10.5152/tpd.2015.3669

73. Bothale KA, Kolhe H, Mahore SD, Wilkinson AR. Diagnosis of primary hydatid cyst of thigh by fine needle aspiration cytology. Indian J Med Microbiol. 2015;33: 151–153. doi:10.4103/0255-0857.148426

74. Demir G, Tekin A, Tekin H. Diagnosis and excision of a primary hydatid cyst localized in the soft tissue. Rev Soc Bras Med Trop. 2014;47: 537. doi:10.1590/0037-8682-0058-2014

75. Aydin BK, Acar MA, Sumer S, Demir NA, Erkocak OF, Ural O. Primary hydatid disease of brachialis and biceps brachii muscles: a case report. Trop Doct. 2014;44: 53–55. doi:10.1177/0049475513512636

76. Ekşi MŞ, Bayri Y, Saraçoğlu A, Uyar Bozkurt S, Konya D. Primary subcutaneous hydatid cyst over thoracic spine: a case report and review of the literature. Turk Parazitolojii Derg. 2014;38: 264–269. doi:10.5152/tpd.2014.3198

77. Ouakrim R, Amziane Z, younes O, Eloukili I, Kharmaz M, Lamrani MO, et al. Locally recurrent subcutaneous and muscular hydatid cysts of the leg: an unusual case report. Pan Afr Med J. 2015;21: 282. doi:10.11604/pamj.2015.21.282.6767

78. Suffee T, Chader H, Foulet F, Herruela C, Djabbari M, Chosidow O, et al. A Suspicious Subcutaneous Tumor. Clin Infect Dis. 2015;61: 1759–1760. doi:10.1093/cid/civ662

79. Spartalis E, Moris D, Athanasiou A, Dimitroulis D, Tomos P. Facing the Unexpected: Chest Wall Swelling 7 Years After A Stab Injury. J Emerg Med. 2016;50: e33–e35. doi:10.1016/j.jemermed.2015.06.023

80. Aït-Ammar N, Prigent G, Zehou O, Le Mouel S, Chader H, Botterel F. [Primary muscle hydatid cyst: About 3 cases]. Presse Medicale Paris Fr 1983. 2016;45: 258–261. doi:10.1016/j.lpm.2015.10.021

81. Hasanoglu I, Aytekin MN, Agackiran Y, Ugurlu M, Guner R, Tosun N. Don't miss it, it might be a hydatid cyst in the gluteus. Trop Doct. 2017;47: 63–65. doi:10.1177/0049475516651186

82. Rodrigues G, Prabhu R. Primary Subcutaneous Hydatid Cyst of the Thigh: An Unusual Site With a Diagnostic Dilemma. J Cutan Med Surg. 2016;20: 266–268. doi:10.1177/1203475415623778

83. Lemrhari B, Baha H, Azzouzi S, Chiheb S. Kyste hydatique sous cutané isolé: à propos d'un cas et revue de la literature. Pan Afr Med J. 2016;24: 212. doi:10.11604/pamj.2016.24.212.9744

84. Moravvej H, Haghighatkhah HR, Abdollahimajd F, Aref S. Primary subcutaneous hydatid cyst of the leg: An unusual location and review of the literature. Indian J Dermatol Venereol Leprol. 2016;82: 558. doi:10.4103/0378-6323.182807

85. Muratori F, De Gori M, D'Arienzo A, Bettini L, Roselli G, Campanacci DA, et al. Hydatid cyst in the vastus lateralis muscle: a case report. Clin Cases Miner Bone Metab Off J Ital Soc Osteoporos Miner Metab Skelet Dis. 2017;14: 262–264. doi:10.11138/ccmbm/2017.14.2.262

86. Ramos Pascua L, Santos Sanchez JA, Samper Wamba JD, Alvarez Castro A, Rodriguez Altonaga J. Atypical image findings in a primary subcutaneous hydatid cyst in the gluteal area. Radiogr Lond Engl 1995. 2017;23: e65–e67. doi:10.1016/j.radi.2017.03.017

87. Al-Hakkak SMM. Adductor magnus muscle primary hydatid cyst rare unusual site: A case report. Int J Surg Case Rep. 2018;51: 379–384. doi:10.1016/j.ijscr.2018.09.026

88. Colazo-Burlato M, Alvarez-Lario B. Muscular Hydatidosis Mimicking Lipoma. J Rheumatol. 2018;45: 1602. doi:10.3899/jrheum.171126

89. Mughal A, Minhas MS, Bhatti A, Moghul FA, Sangani MM, Khan KM, et al. Hydatid Cyst of Skeletal Muscle Presenting as Soft Tissue Tumour. J Coll Physicians Surg--Pak JCPSP. 2018;28: S51–S53. doi:10.29271/jcpsp.2018.03.S51

90. García-Tirado J, Allué-Cabañuz M, Muñoz-González N, Viso-Soriano MJ, Marquina-Ibáñez I. Cystic lesion of pectoralis minor muscle: learning from mistakes. Autopsy Case Rep. 2019;9: e20180780. doi:10.4322/acr.2018.078

91. Salehi H, Salimi Boroujeni K, Yaghoubi S. Report of a Hydatid Cyst Case with Biceps Brachii Involvement. Adv Biomed Res. 2019;8: 23. doi:10.4103/abr.abr_101_18

92. Kurz K, Schwabegger A, Schreieck S, Zelger B, Weiss G, Bellmann-Weiler R. Cystic echinococcosis in the thigh: a case report. Infection. 2019;47: 323–329. doi:10.1007/s15010-018-1255-9

93. Khullar G, Agarwal D, Chandra M. Solitary subcutaneous nodule in the preauricular region. Int J Dermatol. 2020;59: 173–174. doi:10.1111/ijd.14557

94. Grandjean Lapierre S, Barkati S, Desjardins M, Ndao M, Libman M, Poirier L, et al. Isolated muscular cystic echinococcosis mimicking neoplasia. J Travel Med. 2020;27: taaa002. doi:10.1093/jtm/taaa002

95. Ewnte B. Hydatid cyst of the foot: a case report. J Med Case Reports. 2020;14: 6. doi:10.1186/s13256-019-2337-8

96. Mateo L, Roure S, Prior Á, Torres MCS, Subirats JL, Hermoso JAH. Multiple Primary Muscular Hydatidosis in an Immunosuppressed Patient. J Clin Rheumatol Pract Rep Rheum Musculoskelet Dis. 2021;27: S603–S606. doi:10.1097/RHU.0000000000000954

97. Ben Khalifa M, Ghannouchi M, Hammouda S, Taboubi W, Omri A, Nacef K, et al. Primary subcutaneous hydatid cyst: An exceptional location. IDCases. 2022;30: e01627. doi:10.1016/j.idcr.2022.e01627

98. Condor P, Argandoña J, Flores-Lovon K, Ticona D, Cuba-Cáceres N, Gutierrez EL. Primary muscular hydatidosis in an endemic area of Peru: report of two cases. Ann Parasitol. 2022;68: 631–635. doi:10.17420/ap6803.470

99. Arian M, Kazerani M. Primary hydatid cyst in the adductor muscles of thigh: A case report. Clin Case Rep. 2022;10: e6664. doi:10.1002/ccr3.6664

100. Patmano M, Çetin DA, Gümüş T, Patmano G, Yenigül AE. Primary Soft Tissue Hydatid Cysts. Turk Parazitolojii Derg. 2022;46: 145–149. doi:10.4274/tpd.galenos.2021.03511

101. Agholi M, Heidarian HR, Montaseri Z, Khajeh F. Muscular hydatid cyst in Iran: A case report. Int J Surg Case Rep. 2023;103: 107867. doi:10.1016/j.ijscr.2022.107867
